# Commentary: Evaluation of the causal effects of immune cells on ischemic stroke: a Mendelian randomization study

**DOI:** 10.3389/fimmu.2024.1450086

**Published:** 2024-10-23

**Authors:** Xingcheng Zhu, Junhao Chen, Junxian Zhao

**Affiliations:** ^1^ Department of Clinical Laboratory, The Second People’s Hospital of Qujing City, Qujing, Yunnan, China; ^2^ Department of Urology, The Second Affiliated Hospital of Kunming Medical University, Kunming, Yunnan, China; ^3^ Department of Urology, 920th Hospital of Joint Logistics Support Force of Chinese People’s Liberation Army, Kunming, Yunnan, China

**Keywords:** immune cells, ischemic stroke, Mendelian randomization, reverse, sample overlap

## Introduction

We read with great interest the paper by Wang et al. (1), which provides a comprehensive analysis using genome-wide association study (GWAS) data to evaluate the causal impact of various immune cell traits on ischemic stroke (IS). By employing Mendelian randomization, the authors identified several significant associations between immune cell traits and the risk of IS. Notably, they highlighted the role of CD62L-plasmacytoid dendritic cells (pDCs) as a risk factor and CD4+ CD8dim T cells as a protective factor. Their findings underscore the complexity of immune interactions in IS and propose potential biomarkers for early diagnosis and therapeutic targets. However, we believe there are aspects of their methodology and study design that warrant further discussion and improvement.

## Discussion

Firstly, the use of both discovery and validation cohorts is commendable as it acknowledges the specificity of data sets for this category of diseases. However, Wang et al.’s study utilized two data sets from the EBI database (ebi-a-GCST90018864 and ebi-a-GCST005843) in both the discovery and validation phases. This approach raises significant concerns about potential sample overlap between these cohorts. Such overlap could compromise the independence of the validation process, thereby undermining the robustness and reliability of the results. To strengthen the validation phase, it would be beneficial to use entirely independent data sets from different sources. For example, the authors could consider additional data sets from the IEU OpenGWAS project, the FinnGen database, or sources like ieu-a-1108. Utilizing these alternative sources would ensure more stringent and independent validation, enhancing the credibility of the meta-analysis results.

Secondly, the study performed a high-throughput two-sample Mendelian randomization involving 731 immune cell traits with IS. This large-scale analysis inherently increases the risk of false-positive findings due to multiple testing. Applying multiple test corrections, such as False Discovery Rate (FDR) or Bonferroni correction, is crucial to control for these potential errors (2). Without such corrections, there is a greater likelihood of identifying statistically significant associations by chance alone. Implementing FDR or Bonferroni corrections would ensure that the findings are more reliable and reduce the chances of reporting spurious associations. This step is vital in large-scale genomic studies to maintain the integrity of the results and provide more accurate insights into the causal relationship between immune cell traits and ischemic stroke.

Thirdly, incorporating reverse MR analysis could enrich the study by assessing whether IS also leads to changes in immune cell traits, such as increases or decreases in specific cell types. This would provide a more comprehensive understanding of the disease mechanisms. Additionally, reverse MR analysis would help rule out the possibility of bidirectional causality, ensuring that the observed associations are not a result of IS influencing immune cell traits. By confirming that the relationship is unidirectional, the study’s conclusions would be more robust and reliable.

Lastly, we recommend expanding the analysis through multivariable Mendelian randomization (MVMR) and transcriptomics (3). MVMR would allow the authors to assess the independent impact of pDCs and CD4+ CD8dim T cells on IS outcomes while considering them simultaneously. This approach could reveal whether each cell type has a unique contribution to the disease or if their effects are interdependent. Furthermore, after converting SNPs to genes, further analysis using the GEO database should be performed (4). Gene enrichment and KEGG pathway analysis could be conducted to explore the specific biological pathways through which these immune cells influence IS and identify the key genes involved. Integrating these transcriptomic insights with Mendelian randomization results would provide a more comprehensive understanding of the causal mechanisms and validate the findings from multiple perspectives.

## Conclusion

Incorporating validation using diverse data sources and adopting these advanced methodologies will enable a richer and more detailed exploration of the relationship between immune cells and ischemic stroke. This, in turn, will enhance the credibility of the findings related to therapeutic targets and biomarkers.
